# Waiting longer, feeling fatter: Effects of response delay on tactile distance estimation and confidence in females with anorexia nervosa

**DOI:** 10.1002/brb3.2422

**Published:** 2021-11-28

**Authors:** Manja M. Engel, Stephen Gadsby, Andrew W. Corcoran, Anouk Keizer, H. Chris Dijkerman, Jakob Hohwy

**Affiliations:** ^1^ Dijkermanlab Department of Experimental Psychology Faculty of Social and Behavioural Sciences Heidelberglaan 1 Utrecht 3584 CS The Netherlands; ^2^ Cognition and Philosophy Lab Department of Philosophy Monash University 20 Chancellors Walk Clayton Victoria 3800 Australia

**Keywords:** anorexia nervosa, body image, body representation, confidence, tactile distance estimation

## Abstract

**Background:**

Research suggests that patients with anorexia nervosa (AN) exhibit differences in the perceptual processing of their own bodies. However, some researchers suggest that these differences are better explained with reference to non‐perceptual factors, such as demand characteristics or emotional responses to the task. In this study, we investigated whether overestimation of tactile distances in participants with AN results from differences in tactile processing or non‐perceptual factors, by measuring the role of allowed response time in an adapted version of the tactile distance estimation task (TDE‐D). We further investigated the relationship between allowed response time and participants' confidence in their tactile judgments.

**Method:**

Our sample consisted of females: participants with AN (*n* = 30), recovered (REC) participants (*n* = 29) and healthy controls (HC) (*n* = 31). Participants were asked to estimate tactile distances presented on the skin of either a salient (abdomen) or non‐salient (arm) body part, either directly after stimulus presentation (direct condition) or after a 5 s delay (delayed condition). Confidence of estimation accuracy was measured after each response.

**Results:**

Results showed that allowing AN and REC more time to respond caused them to estimate tactile distances as larger. Additionally, participants with AN became less confident when given more time to respond.

**Conclusions:**

These results suggest that non‐perceptual influences cause participants with AN to increase their estimates of tactile distances and become less certain of these estimates. We speculate that previous findings—where participants with AN estimate tactile distances as larger than HC—may be due to non‐perceptual differences.

## Graphical abstract

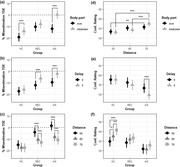

## 1 INTRODUCTION

Patients with anorexia nervosa (AN) display an extreme fear of gaining weight while being (severely) underweight. In addition, AN is characterized by body image disturbance (BID) (American Psychiatric Association, 2013)—a crucial feature of the disorder, associated with both maintenance and onset, and shown to be a predictor for relapse (Carter et al., 2004; Stice & Shaw, 2002).

BID is considered to consist of two components: attitudinal, patients profess to holding negative attitudes toward their own body size, and perceptual, patients overestimate their own body size (Cash & Deagle, 1997). Recently, the perceptual component has attracted significant attention, with researchers discovering that patients with AN overestimate their own body size in several domains. For example, not only do patients visually judge their bodies as larger than reality, they also perceive affordances, and move as if their bodies were larger than reality (Engel & Keizer, 2017; Gadsby, 2017; Guardia et al., 2010, 2012; Keizer et al., 2013; Metral et al., 2014). These biases are generally considered to be specific to the patients’ own bodies, as they are not present when patients are required to estimate the size of other bodies (Guardia et al., 2012), or inanimate objects (Engel et al., 2020; Smeets, 1997). Many of these differences have also been found in recovered (REC) patients (Engel & Keizer, 2017).

Apart from differences in visual size estimation, affordance perception, and movement, patients with AN appear to perceive touch as if their bodies were larger than reality (Keizer et al., 2011, 2012; Risso et al., 2020; Spitoni et al., 2015, cf. Mergen et al., 2018). Specifically, research shows that patients with AN estimate tactile distances as larger than healthy controls (HC). This difference is more pronounced on body parts that are salient in AN (e.g., abdomen, waist) compared to those that are not (e.g., sternum), and on the horizontal rather than vertical axis (Risso et al., 2020; Spitoni et al., 2015). The standard interpretation of these findings attributes them to perceptual differences between patients with AN and HC. For example, one hypothesis claims that, in the case of AN, tactile signals are mapped onto a representation of skin surface which is distorted in ways stereotypical of an overweight body (wider along the thighs and waist). This distortion causes patients to experience distances as wider on salient body parts, along the horizontal axis (Spitoni et al., 2015; Gadsby, 2017; cf. Keizer, et al., 2012).

One point of contention within BID research is how to interpret evidence that patients with AN overestimate their own body size. While many assume that overestimation stems from misperception of the body, an alternative interpretation is that overestimation stems from differences in cognitive and evaluative attitudes regarding body size (Smeets, 1997). This issue has plagued BID research since its emergence in the 1980s (Ben‐Tovim et al., 1990), and the debates are still ongoing (Cornelissen et al., 2019; Mölbert et al., 2018; Wignall et al., 2017). The issue also problematizes findings from tactile perception research. Rather than perceiving tactile distances to be wider, attitudinal factors may cause patients with AN to *estimate* tactile distances as wider than HC. These factors could take many forms, for example: patients’ belief that certain body parts are overweight, their emotions associated with certain body parts (Øverås et al., 2014), or demand characteristics involved in the setup (i.e., patients’ beliefs about how the experimenter desires them to perform) (Proctor & Morley, 1986).

A fruitful way in which perceptual and attitudinal influences on body size estimation can be experimentally teased apart is through the manipulation of allowed response time. For example, while intuitive responses based off perception occur quickly, cognitive reasoning takes longer (Rubinstein, 2007). In all previous studies that found differences in tactile distance estimation between AN and HC, responses were self‐paced: participants were given as much time as they desired to respond (Spitoni, et al., 2015; Keizer et al., 2011, 2012). This design allows participants time to reflect on beliefs and emotional attitudes regarding their own body size, which may bias the response in distance judgment (D'Amour & Harris, 2017). Therefore, these findings may confound perceptual with attitudinal influences.

In addition to evidence suggesting that patients misestimate their own body size, multiple strands of evidence suggest that patients with AN exhibit low confidence in their body attitudes and perceptions. For example, several studies employ semi‐structured interviews to investigate the confidence that patients exhibit in their beliefs about being overweight (Konstantakopoulos et al., 2012; Mountjoy et al., 2014). These results suggest that many patients exhibit low confidence in relation to such beliefs. Other research focuses on the confidence that patients exhibit in their perception of their own bodily states. These results suggest that patients exhibit low confidence in their perception of interoceptive sensations such as hunger and heartbeat (Fassino et al., 2004; Jenkinson et al., 2018; Kinnaird et al., 2020). However, the confidence that patients hold in their tactile experiences of their own bodies has yet to be explored.

This study investigated the extent to which estimates of tactile distances in patients with AN and REC are influenced by non‐perceptual factors, by investigating the role of allowed response time in tactile size estimation. In this design, participants were asked to estimate tactile distances presented on the skin of either a salient (abdomen) or non‐salient (arm) body part, either directly after stimulus presentation (direct condition) or after a 5 s delay (delayed condition). By asking for a direct response, we minimized the opportunity for the cognitive evaluation that can occur during a longer response window (delayed response) (Rubinstein, 2007). We thus tested whether tactile size estimation is, in part, influenced by attitudinal factors. We also included a confidence measure to investigate whether between‐group differences in estimation confidence could be found—in line with the aforementioned proposals regarding eating disorders and confidence in bodily perception and belief.

We expected an influence of delayed response on tactile size estimates in participants with AN. Specifically, we expected delayed estimates to be larger in our AN group, and for this effect to be amplified in salient body parts. However, we expected no differences between direct and delayed conditions for the HC and REC groups, as neither of these groups exhibit negative body attitudes of the same severity as patients with AN (Engel & Keizer, 2017). Over both delayed and direct conditions, we expected REC participants to estimate tactile distances as larger than HC (albeit smaller than AN), consistent with evidence of persistent perceptual dysfunction within this group.

We further expected that participants with AN would report lower confidence ratings on average compared to HC and REC. We also expected group differences in confidence ratings to interact with response delay, such that confidence ratings increase in HC, but decline in AN over time.

## 2 METHOD

### 2.1 Ethics statement

This study was approved by Monash University Human Research Ethics Committee (MUHREC ProjectIDs: 19265 & 19131). All participants provided signed an informed consent before taking part.

### 2.2 Pre‐registration

Experimental hypotheses, methods, and planned analyses were pre‐registered prior to data collection: https://aspredicted.org/tk5c9.pdf. We aimed to recruit 30 participants into each group.

### 2.3 Participants

Participants were females recruited through the Eating Disorders Victoria Facebook page, Twitter, and posters distributed around various higher education institutions in the Melbourne metropolitan area. All participants were compensated for their time.

Participants with AN and REC were included if they had a present or past diagnosis of AN and REC, respectively, obtained from a psychiatrist or general physician. This diagnosis was verified with the EDE‐Q. Our criteria for REC participants were twofold: REC participants self‐reported that they had successfully completed treatment for their eating disorder and that they were no longer in need of treatment. Self‐reported diagnosis was then checked with the EDE‐Q. When the EDE‐Q matched the self‐reported diagnosis, these participants were included in the REC group. HC were included if they self‐reported no history of ED or any other acute diagnosis at the time of testing.

We recruited 98 individuals who fit our inclusion criteria. Eight were excluded as they failed to discriminate two simultaneously presented points on the skin of the arm or abdomen at a distance of 40 mm (Weinstein, 1968). For a thorough description of the two‐point discrimination task, see Supplementary Material. Our final sample consisted of 30 participants with AN, 29 REC participants, and 31 HCs (see Table 1 for demographics).

**TABLE 1:** Demographics, clinical assessment, EDE‐Q, and BAT scores

	HC *n* = 31	REC *n* = 29	AN *n *= 30
**Demographics**			
Age	22.55 ± 3.79	22.76 ± 3.99	22.17 ± 4.14
Age range	19–34	19–35	18–32
BMI	21.06 ± 3.42	21.60 ± 2.74	19.55 ± 3.21
BMI range	16.23–32.37	17.26–30.11	14.15–26.27
Right‐handedness	27	26	28
**Reported diagnosis**			
AN – Restrictive	—	18	28
AN – Binge/purge	—	10	1
OSFED	—	1	1
Other lifetime diagnoses	—	27	28
OSFED	—	1	1
Other diagnoses	—	27	28
Age: symptom onset	—	13.90 ± 2.58	13.97 ± 3.10
Age: AN diagnosis	—	16.14 ± 2.52	17.23 ± 2.98
Age: start of treatment	—	14.72 ± 5.74	14.47 ± 8.18
Duration treatment	—	2.48 ± 2.79	3.77 ± 3.78
**EDE‐Q**			
**Global score**	1.17 ± 0.99	1.86 ± 1.22	**3.33 ± 1.35**
**Restraint**	0.80 ± 1.03	1.26 ± 1.19	**3.16 ± 1.79**
**Eating concern**	0.62 ± 1.02	1.38 ± 1.22	**2.78 ± 1.59**
**Shape concern**	1.83 ± 1.26	2.55 ± 1.54	**3.94 ± 1.33**
**Weight concern**	1.41 ± 1.32	2.25 ± 1.38	**3.45 ± 1.42**
**BAT**			
**Total score**	23.00 ± 16.83	**35.93 ± 13.90**	**47.40 ± 13.33**
**Negative appreciation**	7.32 ± 6.19	13.14 ± 6.09	**16.80 ± 6.36**
**Lack of familiarity**	7.68 ± 4.52	**12.28 ± 5.12**	**17.53 ± 6.15**
**General dissatisfaction**	8.00 ± 4.16	10.52 ± 4.19	**13.07 ± 3.65**

*Note*: For a full overview of other lifetime diagnoses, see Table S.1. Duration of treatment is in years (*± SD*). Boldface indicates significant difference from corresponding estimate in the preceding column.

### 2.4 Materials and procedure

After providing signed, informed consent, participants completed a demographic questionnaire and the following questionnaires and tasks in the order they are listed below.

#### 2.4.1 Eating Disorder Examination Questionnaire

The Eating Disorder Examination Questionnaire (EDE‐Q) (Fairburn & Beglin, 1994) was used to further substantiate self‐reported diagnosis of participants with AN and check for ED pathology in HC. The EDE‐Q is a widely used, validated, self‐report instrument to assess ED psychopathology (Aardoom et al., 2012). This approach (self‐report, justified by the EDE‐Q) has been used by other studies to distinguish clinical groups (Berg et al., 2011; Black & Wilson, 1996; Fairburn & Beglin, 1994; Anderson & Williamson, 2002). The validity and discriminability of the EDE are good, and the EDE‐Q scores and Eating Disorder Examination (EDE) interview (clinical diagnostic interview for ED) are highly correlated (see Mond et al. (2004)). The EDE‐Q is recommended as a replacement of the EDE interview (Berg et al., 2011; Black & Wilson, 1996; Fairburn & Beglin, 1994; Anderson & Williamson, 2002).

Global scores in our sample were compared against normative data of a large community sample of Australian adult women (Mond et al., 2004). Higher scores reflect higher levels of eating disorder pathology.

#### 2.4.2 Body attitude test

The body attitude test (BAT) (Probst et al., 1995) was used to compare the extent of negative body attitudes between groups. The BAT was developed for female patients suffering from eating disorders and consists of four subscales: "negative appreciation of body size," "lack of familiarity with one's own body," "general body dissatisfaction," and a rest factor. The total score across these scales is indicative of body attitudes, with higher scores reflecting more negative attitudes.

#### 2.4.3 Tactile Distances Estimation Questionnaire

The Tactile Distances Estimation Questionnaire (TDE‐Q) was especially designed for this study to measure individuals’ attitudes toward their own body parts. The TDE‐Q consists of four questions; two questions tapping the evaluation of the salient (abdomen) and non‐salient (arm) body part, another two questions of how this feeling varies over time. For example, regarding the salient body part, participants were asked "*How do you feel about the size of your abdomen?*," responding on a visual analogue scale (VAS) ranging from "*too thin"* to "*too fat*." To assess variation over time, they were asked "*Does your evaluation of the size of your abdomen change during the day?"* Again, participants answered on a VAS with "*it is stable"* and "*it varies all the time*" as anchors. Responses were measured on a 0–100 scale. Note, the TDE‐Q has not been formally validated.

#### 2.4.4 Tactile distance estimation–delayed

An adapted version of the TDE task (Keizer et al., 2011) was developed to manipulate the time delay between tactile stimulation and estimation. In this tactile distance estimation–delayed (*TDE‐D*) task, participants were presented with two tactile points applied with a caliper on the skin of the left forearm (non‐salient), on the proximo‐distal axis, or the left side of the abdomen (salient), on the medial‐lateral axis. Participants were asked to estimate the distance between these two points by placing the index finger and thumb of their right hand on a tablet. Participants made their estimate when they heard a sound cue (10,000 Hz tone). This cue was played directly after stimulus presentation (direct) or 5 s post‐stimulus (delayed).

**FIGURE 1:**
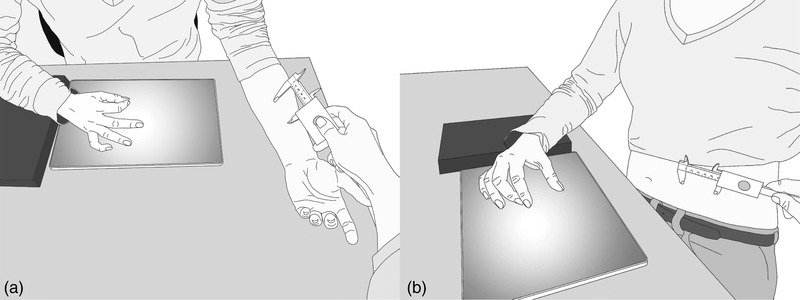
Set up of estimates on (a) arm and (b) abdomen. A caliper was used to present distances. The experimenter pressed a button on the caliper that was connected to an earpiece where an audio sound was played for 300 ms (duration of stimulus presentation).

Participants were instructed to close their index finger and thumb before each trial, and to respond as soon as they heard the sound. Practice trials were performed until participants responded directly on the audio cue. The right arm was placed on an elevated surface with the wrist above the tablet so that estimations could be made quickly (see Figure 1). Distances of 50, 60, and 70 mm were presented in a randomized order (total 5 trial repetitions per distance) for each body‐part (salient, non‐salient) and response‐delay (direct, delayed) condition. The order of the response‐delay and body‐part condition blocks was counterbalanced across participants. All tactile stimuli were presented for 300 ms. The experimenter maintained presentation time consistency by applying stimulation concurrent with a 300 ms tone played to them through an earpiece.

#### 2.4.5 Confidence rating

Our confidence questionnaire was hosted on Gorilla Experiment Builder (www.gorilla.sc). After each distance estimate, participants were asked *"How confident are you that your estimate is correct?"* They responded on a VAS, with "total guess" anchoring a rating of 0, and "complete confidence" anchoring a rating of 100.

### 2.5 Data preparation and analysis

#### 2.5.1 Planned analysis

EDE‐Q subscale scores were derived by averaging item scores; EDE‐Q global score was calculated from the average of subscale scores. BAT items were summed to derive subscale and total scores. Mixed ANOVAs were conducted in IBM SPSS Statistics for Windows, Version 25.0, to test between‐subjects differences in EDE‐Q and BAT scores. A mixed ANOVA was used to test for between‐group differences in mean VAS scores on each item of the TDE‐Q. The *p*‐values for planned comparisons were Bonferroni corrected.

TDE‐D and confidence tasks were modeled using mixed ANOVAs, with group (AN; REC; HC) included as a between‐subjects independent variable, and body‐part (arm; abdomen), distance (50, 60, and 70 mm), and response‐delay (direct; delayed) as within‐subjects independent variables. In order to facilitate comparison of estimation accuracy across distance levels, we normalized estimates using the following formula:

Percentage misestimation = (Estimated distance − Actual distance)/Actual distance*100.

One‐tailed planned comparisons were used to test prespecified hypotheses. Missing data were handled by list‐wise deletion.

Shapiro‐Wilk tests and data plots were used to assess normality. Levene's test was used to assess homogeneity of variance. Where this test indicated heteroskedasticity, *Welch's F* was computed (Field, 2009). Mauchly's test was conducted for all mixed ANOVAs. Where this test indicated violation of the sphericity assumption, the Greenhouse–Geisser correction was used for epsilons ranging from 0.50 to 0.75, otherwise the Huynh–Feldt correction was used (Field, 2009).

#### 2.5.2 Additional analysis

In addition to our planned analyses, we fit linear mixed‐effects models (LMMs) to model distance estimation and confidence at the trial level. These analyses were performed on account of the large degree of individual variation observed within the dataset, and to mitigate loss of information due to list‐wise deletion of missing data. In order to replicate the structure of our planned ANOVAs, random effects were limited to by‐participant random intercepts (more complex models are included in the supplementary materials). The key advantages of this approach over traditional ANOVA are (1) increased power on account of trial‐level estimation and (2) "partial‐pooling" of information across individual and group terms. Together, these innovations improve the accuracy and reliability of parameter estimates (Gelman & Hill, 2006).

LMM analyses were conducted in *R* (v3.6.2; R Core Team, 2019) with *RStudio* (v1.2.5033; RStudio Team, 2015). LMMs were fit using the package *lme4* (Bates et al., 2015). Diagnostic plots revealed no evidence of violated assumptions. Although distance can be construed as a random effect, the low number of sampled levels led us to include it as an ordered factor (polynomial contrasts); all remaining factors were unordered and sum‐to‐zero contrast‐coded. Main effect and interaction terms were assessed using Kenward‐Roger F tests (Satterthwaite degrees of freedom) from Type‐II ANOVA tables obtained from the *car* package (Fox & Weisberg, 2018a). Planned (Bonferroni‐corrected) and post hoc (Tukey‐corrected) comparisons were evaluated using the *emmeans* package (Lenth, 2021). *Effects* (Fox & Weisberg, 2018a, 2018b) and *ggplot2* (Wickham, 2016) were used to visualize model predictions (visualizations of response distributions are included in the supplementary materials).

## 3 RESULTS

### 3.1 Planned analysis

#### 3.1.1 EDE‐Q

A mixed ANOVA showed significant between‐group effects for global EDE‐Q score, *F* (2,87) = 26.20, *p *< .001, *η^2^ *= .37. Significant differences were also found for all subscales: Restraint, *Welch's F* (2,55.42) = 25.09, *p *< .001, *ω *= .30; Eating concern, *Welch's F* (2,55.83) = 21.81, *p *< .001, *ω *= .30; Shape concern, *F* (2,87) = 18.49, *p *< .001, *η^2^ *= .30; Weight concern, *F*(2,87) = 17.04, *p *< .001, *η^2^ *= .28. Post hoc comparisons indicate that our AN sample demonstrated a higher level of ED psychopathology than the REC and HC groups, which is consistent with the self‐reported diagnosis. For means, standard deviations and post hoc comparisons, see Table 1.

#### 3.1.2 BAT

A mixed ANOVA showed significant between‐group differences for total BAT score, *F* (2,87) = 24.54, *p *< .001, *η^2^ *= .36. Significant differences were also found for all subscales: Negative appreciation with one's own body size, *F* (2,87) = 18.06, *p *< .001, *η^2^ *= .29; Lack of familiarity with one's own body, *F* (2,87) = 26.40, *p *< .001, *η^2^ *= .38; General body dissatisfaction, *F* (2,87) = 12.19, *p *< .001, *η^2^ *= .22. Post hoc comparisons indicate that, overall, AN and REC participants have more negative body attitudes than HC, and that negative body attitudes (apart from negative appreciation) are higher in participants with AN than REC participants. For means, standard deviations, and post hoc comparisons, see Table 1.

#### 3.1.3 TDE‐Q

A mixed ANOVA showed significant between‐group differences for evaluation of arm size, *F* (2,87) = 3.88, *p *< .05, *η^2^ *= .08. Post hoc comparisons revealed that participants with AN evaluate their arm as more fat (*M *=* *60.30, *SD *= 22.09) compared to HC (*M *=* *46.90, *SD *= 5.77) (*p <* .05). No differences were found between AN and REC participants (*M *=* *56.07, *SD *= 19.83) (*p *=* *1.0), or REC participants and HC (*p *= .21).

Significant differences were also found for change in evaluation of arm size during the day, *Welch's F* (2,51.93) = 4.72, *p *< .05, *ω *= .07. Participants with AN (*M = *33.47, *SD *= 38.15) report more fluctuation during the day compared to HC (*M = *11.74, *SD *= 16.73) (*p *< .05). No significant differences were found between AN and REC participants (*M = *18.72, *SD *= 25.47 (*p *= .14), or REC participants and HC (*p *= 1.0).

Significant differences were also found for change in evaluation of abdomen size during the day, *F* (2,87) = 13.65, *p *< .001, η^2^ = .24. Participants with AN (*M *= 79.10, *SD *= 28.47) and REC participants *(M *=* *65.59, *SD *= 26.98) report more fluctuation compared to HC (*M *=* *42.74, *SD *= 27.03; *p *< .001 and < .05, respectively). No differences were found between AN and REC participants (*p *= .19).

No significant differences were found for evaluation of abdomen size, *F* (2,87) = 2.82, *p *= .065.

Taken together, these results imply that participants with AN rate their arm as fatter compared to REC participants and HC. Participants with AN also report more change in their evaluation of arm and abdomen size compared to HC. The latter was also apparent in REC participants compared to HC.

#### 3.1.4 TDE‐D

Two participants were excluded from analysis due to missing data (technical error). The full ANOVA table is presented in Table 2 (see Table S2 for descriptive statistics).

**TABLE 2:** Planned analysis: ANOVAs for TDE‐D and confidence ratings

Source	*df*	*F*	*p*	*Partial η^2^*
**TDE‐D**				
**Body‐part**	**1, 85**	**10.68**	**.002**	**.11**
**Response‐delay**	**1, 85**	**14.63**	**< .001**	**.15**
**Distance**	**1.46, 170** ^a^	**26.83**	**< .001**	**.24**
Group	1, 85	2.09	.13	
Group*Distance	**4, 85**	**4.30**	**.002**	**.09**
Group*Body‐part	2, 85	1.77	.18	
Group*Response‐delay	2, 85	1.64	.20	
Body‐part*Response‐delay	1, 85	.84	.36	
Body‐part*Distance	2, 170	1.81	.17	
Response‐delay*Distance	1.90, 170^b^	2.76	.07	
Body‐part*Response‐delay*Group	2, 85	.53	.59	
Body‐part*Distance*Group	2, 85	.55	.70	
Response‐delay*Distance*Group	4, 85	.52	.72	
Body‐part*Response‐delay*Distance	1.91, 170^b^	.42	.65	
Body‐part*Response‐delay*Distance*Group	8, 85	.42	.80	
**Confidence r atings**				
**Distance**	**1, 79**	**13.66**	**<.001**	**.15**
Body‐part	1, 79	.024	.876	
Response‐delay	1, 79	3.00	.087	
Group	1, 79	1.40	.254	
Distance*Group	**4, 158**	**3.16**	**.016**	**.07**
Distance*Body‐part	**1.93, 152.48** ^b^	**4.94**	**.009**	**.06**
Group*Body‐part	2, 79	.012	.989	
Group*Response‐delay	2, 79	2.12	.127	
Body‐part*Response‐delay	1, 79	.481	.490	
Response‐delay*Distance	1.84, 145.15^b^	.69	.49	
Body‐part*Response‐delay*Distance	1.95, 153.9^b^	1.72	.183	
Body‐part*Response‐delay*Group	2, 79	.25	.777	
Body‐part*Distance*Group	4, 158	1.86	.120	
Response‐delay*Distance*Group	4, 158	1.97	.101	
Body‐part*Response‐delay*Distance*Group	4, 158	.87	.486	

^a^ Greenhouse–Geisser correction.

^b^ Huynh–Feldt correction.

Participants with AN's performance on the TDE‐D during the Salient condition did not significantly differ as a function of response‐delay compared to REC participants and HC, *F* (1,85) = 1.104, *p *= .296. REC participants and HC also showed no significant difference in estimation accuracy on this contrast, *F* (1,85) = 1.950, *p *= .166. TDE‐D did not significantly differ between groups, *F* (1,85) = 1.968, *p *= 164.

#### 3.1.5 Confidence ratings

Eight participants were excluded from analysis due to missing data (technical error). The full ANOVA table is presented in Table 2 (see Table S3 for descriptive statistics).

TDE‐D confidence ratings did not significantly differ between participants with AN and REC participants or HC (*F* (1,79) = 2.758, *p *= .101), nor REC participants and HC (*F* (1,79) = 0.055, *p *= .815). As predicted, AN participants’ confidence ratings declined significantly from the direct to the delay condition, *F* (1,79) = 4.194, *p *< .05. However, response‐delay did not significantly modulate confidence ratings in HC, *F* (1,79) = 1.574, *p *= .213.

### 3.2 Additional analysis

#### 3.2.1 TDE‐D

The ANOVA table from the random‐intercepts LMM of TDE‐D estimates is presented in Table 3 (see Table S4 for model summary).

**TABLE 3:** Additional analysis: LMMs for TDE‐D and confidence ratings

Source	*df*	*F*	*p*
**TDE‐D**			
**Body‐part**	**1, 5210.6**	**103.32**	**<.001**
**Response‐delay**	**1, 5210.6**	**54.98**	**<.001**
**Distance**	**2, 5210.0**	**50.79**	**<.001**
Group	2, 87	2.12	.126
Group*Body‐part	**2, 5210.6**	**18.40**	**<.001**
Group*Response‐delay	**2, 5210.6**	**7.03**	**<.001**
Group*Distance	**4, 5210.0**	**6.59**	**<.001**
Body‐part*Response‐delay	1, 5210.7	1.61	.205
Body‐part*Distance	2, 5210.0	1.65	.192
Response‐delay*Distance	2, 5210.0	2.74	.064
Group*Body‐part*Response‐delay	2, 5210.0	0.94	.39
Group*Body‐part*Distance	4, 5210.0	0.57	.68
Group*Response‐delay*Distance	4, 5210.0	0.60	.66
Body‐part*Response‐delay*Distance	2, 5210.0	0.38	.68
Group*Body‐part*Response‐delay*Distance	4, 5210.0	0.19	.94
**Confidence r atings**			
**Distance**	**2, 5037**	**26.44**	**<.001**
**Response‐delay**	**1, 5039.7**	**15.96**	**<.001**
Body‐part	1, 5041.4	0.46	.497
Group	2, 86.0	1.57	.215
Body‐part*Distance	**2, 5037**	**4.55**	**.011**
Group*Response‐delay	**2, 5039.8**	**7.52**	**< .001**
Group*Distance	**4, 5037**	**4.33**	**< .001**
Group*Body‐part	2, 5041.4	0.74	.477
Body‐part*Response‐delay	1, 5039.8	0.23	.633
Response‐delay*Distance	2, 5037	1.87	.155
Group*Body‐part*Response‐delay	2, 5039.8	0.05	.949
Group*Body‐part*Distance	4, 5037	0.99	.410
Group*Response‐delay*Distance	4, 5037	1.28	.277
Body‐part*Response‐delay*Distance	2, 5037	0.82	.441
Group*Body‐part*Response‐delay*Distance	4, 5037	0.60	.665

TDE‐D in AN did not significantly interact with response‐delay and body‐part, *t* (5210) = 1.04, *p *= .650. In the salient condition, delay‐induced changes in estimation performance did not significantly differ between AN and REC participants, *t* (5210) = 0.40, *p *= 1, but did significantly differ between REC participants and HC, *t* (5210) = 6.45, *p *= .008. No differences were found in the non‐salient condition (*p*s > .09). Modeling group as a linear trend supported the hypothesis that TDE‐D estimates were largest for AN and smallest for HC, *t* (93.54) = 2.01, *p *= .048.

**FIGURE 2:**
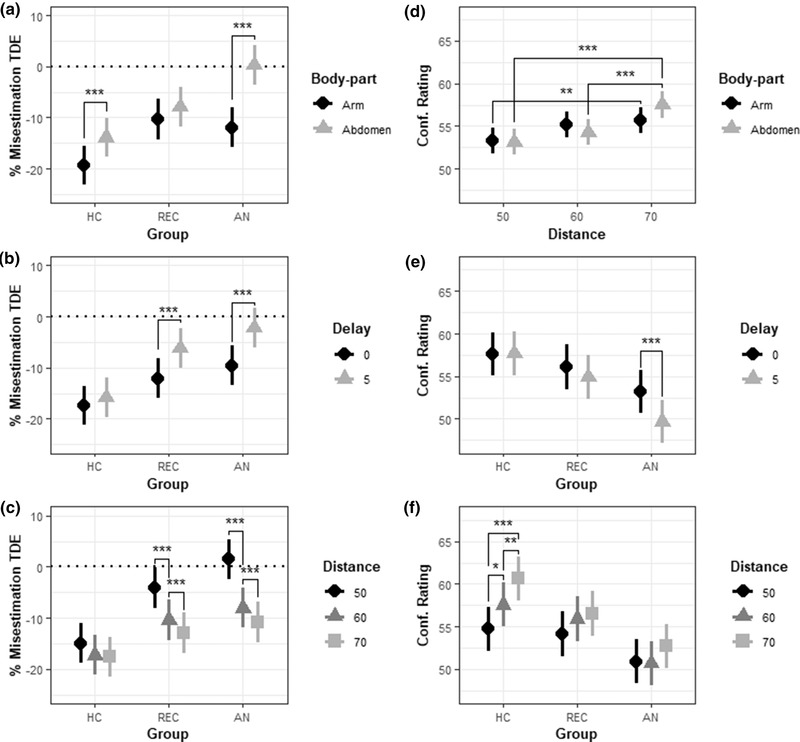
Significant interactions from TDE‐D (left column) and confidence ratings (right column) LMMs. (a) Group*Body‐part interaction. (b) Group*Delay interaction. (c) Group*Distance interaction. (d) Distance*Body‐part interaction. (e) Group*Delay interaction. (f) Group*Distance interaction. *** = *p* < .001, ** = *p* < .01, * = *p* < .05, error bars depict S.E. *Note*: *p*‐values refer to results of additional analysis

The LMM further revealed significant two‐way interactions between Group*Body‐part, Group*Response‐delay, and Group*Distance. Post hoc contrasts showed that participants with AN and HC underestimated distances less on the abdomen compared to the arm (both *p*s < .001; see Figure 2a). AN and REC participants both estimated distances as larger in the delayed condition compared to the direct condition (*p*s < .001; see Figure 2b), while their distance estimates in the 50 mm condition differed significantly from those in the 60 mm and 70 mm condition (all *p*s < .001; see Figure 2c).

#### 3.2.2 Confidence ratings

The ANOVA table from the random‐intercepts LMM of confidence ratings is presented in Table 3 (see Table S5 for model summary).

TDE‐D confidence ratings did not significantly differ between AN and REC participants, *t* (85.9) = 1.12, *p *= .265, nor REC participants and HC, *t* (86) = 0.60, *p *= .553. As predicted, participants with AN reported lower confidence after a delayed response, *t* (5038) = 5.26, *p *< .001. By contrast, HC showed no significant difference in confidence ratings between direct and delayed trials, *t* (5044) = 0.16, *p *= .44 (see Figure 2e).

The LMM revealed additional significant interactions between Body‐part*Distance and Group*Distance. Post hoc analysis of the Distance*Body‐part interaction revealed that participants were more confident about their estimation accuracy on the arm for 70 mm compared to 50 mm (*p *= .005). On the abdomen, participants were more confident at 70 mm compared to 50 mm (*p *< .001) and at 70 mm compared to 60 mm (*p *< .001; see Figure 2d). Contrasts for the Group*Distance interaction revealed that HC were more confident in distance estimates at 70 mm compared to 60 mm (*p *= .005), 70 mm compared to 50 mm (*p *< .001), and 60 mm compared to 50 mm (*p *= .016; See Figure 2f).

Finally, distance estimates and confidence ratings were correlated with clinical variables: symptom onset, illness duration, duration of treatment, and BMI. No significant results survived Bonferroni correction (alpha = .003). This indicates that the clinical variables and BMI do not influence our outcome measure.

## 4 DISCUSSION

A much‐debated issue in AN research is whether overestimation of body size reflects a difference in perceptual processing, or stems from non‐perceptual factors, such as the cognitive and affective attitudes patients hold toward their own bodies (Smeets, 1997). This study aimed to investigate the comparative influence of perceptual and non‐perceptual factors on tactile size estimation in participants with AN and REC, by manipulating response delay. Here, a delayed response allowed participants to deliberate, increasing the influence of non‐perceptual factors (e.g., attitudes and emotions) on their final estimate, while a direct response did not include this deliberation period, more directly reflecting their perception of the distance. Additionally, we investigated if this response delay affects confidence in the accuracy of these judgments.

We expected a longer response time would increase tactile distance estimates in participants with AN, especially in salient body parts. We also expected REC participants to estimate distances as larger than HC, but smaller than participants with AN. While our planned analyses indicated that tactile distance estimation varied as a function of both body‐part and response‐delay, group‐level interactions were only revealed when we modeled participant responses on the trial‐level, using linear mixed‐effects modeling (LMM). The results of this additional analysis revealed that participants with AN and REC, but not HC, judge distances as larger when responding after a 5 s delay period. Further, tactile distances were judged as larger on the forearm compared to the abdomen in participants with AN and HC (but not REC). Contrary to our expectations, response‐delay did not interact with body‐part salience.

In terms of estimation confidence, we expected participants with AN to report lower confidence than HC and REC. Additionally, we expected participants with AN to be less confident in the delay, compared to the direct condition, while we expected the opposite for HC. Although our ANOVA failed to provide statistical evidence of these effects, the additional sensitivity afforded by the LMM furnished partial support: participants with AN reported lower confidence in their estimation accuracy in the delayed condition, compared to the direct condition. Contrary to our expectations, we did not see an influence of response‐delay on confidence ratings in REC or HC.

We did not discover between group‐differences in tactile distance estimation, in either the direct or delayed conditions (see also: Engel & Keizer, 2017). Consequently, our results do not directly support the claim that non‐perceptual influences cause participants with AN to estimate tactile distances as larger than HC. However, given our finding that AN participants increase their estimates when given more time to respond, we suggest that this mechanism may, in some experimental setups, drive a between‐group effect. We speculate that previous findings of comparative overestimation by participants with AN may be driven by the mechanism uncovered here (non‐perceptual factors increasing estimates, within this group).

Our results also illuminate the non‐perceptual factors that drive the observed finding, causing participants with AN to increase their estimates. Previous research suggested that either attitudes about body size (e.g., beliefs about being overweight) or demand characteristics (beliefs about what the experimenter expects) may cause participants with AN to overestimate their bodies (Smeets, 1997). Such explanations predict that these participants would only increase their estimates (when given more time to respond) in the salient (abdomen, medio‐lateral) as opposed to non‐salient (arm, proximo‐distal) condition. This is because patients with AN do not hold false beliefs about their arm length and would not assume the experimenter expected them to misestimate those dimensions. Our results contradict this prediction, as when looking at the AN group, there is no delay*body‐part interaction (see: Figure S7). Given this finding, whichever non‐perceptual factors drive misestimation, they must affect both body‐part conditions. Explanations of this finding may refer to changes in the memory of the touch experience (Williamson et al., 2004); or judgment biases driven by time to reflect on anxiety (Øverås et al., 2014) or task difficulty (Waller & Hodgson, 1996). While our data cannot distinguish between the various possibilities, this represents a promising avenue for future research.

Another novel finding of the study stems from our use of the TDE‐Q, designed to measure fluctuations in bodily attitudes. The results of this questionnaire suggest that patients with AN show a higher fluctuation in their evaluative attitudes toward their arms and abdomens, compared to HC. Interestingly, this measure also discovered that REC patients’ evaluation of their own abdomen size fluctuates significantly more compared to HC. These daily fluctuations in evaluation of body size are additional indicators of uncertainty in the body size attitudes of patients with AN and REC (Espeset et al., 2011).

In further support of a difference in confidence between AN, REC, and HC, we found that while HC were more confident for larger compared to smaller distances, this was not the case for AN and REC, who exhibited no between‐distance differences in confidence ratings. The results from our HC group can be explained with reference to perceptual ambiguity: smaller tactile distances are closer to the 2‐point discrimination threshold and are thus more perceptually ambiguous, causing participants to be less confident in such judgments. While it is not clear how to explain the homogeneity of confidence judgments in patients with AN and REC, future research might clarify this issue by using more precise confidence measures (Matthews et al., 2020).

Although our findings might be taken to suggest that targeting cognitive‐evaluative attitudes is more clinically effective than targeting bodily perception, it is too early to draw such a conclusion. First, the attitudinal influence present in our delayed condition is still situated within a perceptual context, aspatients’ deliberations are directly prompted by a bodily experience. This is important, as “perceptual” interventions are usually targeted at attitudinal responses to perceptual experience, for example, challenging negative thoughts that arise during self‐viewing (Delinsky & Wilson, 2006). Second, we only measured tactile perception in this study, while perceptual training usually targets other aspects of perception (visual perception, affordance perception) (Keizer et al., 2019). Further research should explore whether errors in perception are present in these domains.

To test and replicate previous findings, we adopted a TDE paradigm employed in Engel & Keizer (2017), Keizer et al. (2011), and Keizer et al. (2012). This introduces an important caveat to our interpretation. Based on our results, we speculated that that AN and REC overestimate due to non‐perceptual factors; however, this may also be specific to the paradigm used here. For example, in visual body size estimate research, the type of measure employed has been found to modulate body size estimates (Cornelissen et al., 2017). Some studies that found differences in TDE between AN and HC used alternative methods (Risso et al., 2020; Spitoni et al., 2015), and these methods may be more suited to capturing perceptual differences between these groups (see also: Tosi & Romano, 2020). Accordingly, future research should further investigate these claims, employing various methods of assessing tactile size perception.

Another caveat to note is that, in increasing their estimates of tactile distances (in the delayed condition), AN and RAN were in fact estimating distances more accurately. This finding is consistent with results from previous research, which show that AN groups are often more accurate than HC in their body size estimates, in virtue of underestimating less (Bowden, et al., 1989; Meermann, 1983; Lindholm & Wilson, 1988; for review and discussion, see Farrell et al., 2005). In the literature on eating disorders, the focus is on the differences in estimates provided by the different groups (AN, REC, HC), not the accuracy of those estimates (Keizer et al., 2011, 2012). The important difference between these groups (BID) provides theoretical reason to assume that AN and REC would estimate distances as larger than HC (Gadsby, 2017). In contrast, there is no clear, theoretically supported reason for why AN and REC would exhibit greater accuracy in their tactile size estimates. Nevertheless, there may still be some explanation for why AN and RAN would become more accurate in their estimates, compared to HC, when given more time to respond. Providing such an explanation is, however, beyond the scope of this discussion.

We acknowledge that the critical interactions between group and response‐time were uncovered in the unplanned component of our analysis. While such findings are usually caveated with the need for cautious interpretation, it should be stressed that our LMMs aimed to emulate the ANOVAs specified in our preregistered analysis with greater power and precision. Although LMMs are commonly regarded in psychology as more complex and difficult to interpret than traditional methods (Meteyard & Davies, 2020), our findings showcase the advantages of such techniques in the context of substantial individual variation and unbalanced data—common occurrences in clinical research settings.

One limitation of our study is that our participant groups do not significantly differ in terms of BMI. Our mean BMI for participants with AN is higher than cut‐off scores for diagnosis. One possible explanation is that participants with AN already started their ED treatment and were already in the process of regaining their lost weight, as weight regain is one of the main focusses in AN treatment (Noordenbos & Elburg, 2018). However, gaining weight does not necessarily resolve BID—in many cases, it worsens them (Cornelissen et al., 2017)—nor the attitudes toward gaining weight. That eating behaviors and body attitudes remain problematic, despite weight gain, is reflected in the results of the EDE‐Q, where participants with AN scored significantly higher than REC and HC on ED psychopathy. These results indicate that core symptoms of AN are still very much present even though their BMI is not significantly different than those of REC and HC. Furthermore, we did not find a correlation between BMI and our TDE scores. In our past studies using the TDE (where we also included clinical samples, where BMI did fall under the weight cut‐off for the diagnosis), we found no correlation between BMI and the outcome measures (e.g., Engel & Keizer, 2017; Keizer et al., 2011, 2012). This is also consistent with other findings of no correlation between low BMI and BIDs (using both attitudinal and perceptual measures) (Mölbert et al., 2018; Ben‐Tovim et al., 1990). This indicates that BMI is not a factor of influence when it comes to overestimation, as opposed to attitudes toward the body (as suggested by our results).

In summary, we showed that allowing participants with AN and REC more time to respond during tactile distance estimation causes them to make larger distance estimates. This finding is consistent with a non‐perceptual explanation of tactile distance overestimation in AN. Based on this discovery, we speculate that previous findings of participants with AN estimating tactile distances as larger than HC may be due to non‐perceptual differences. We also discovered that, in contrast to HC and REC, participants with AN became less confident when given more time to respond, contributing to our knowledge of differences in confidence associated with the disorder.

### PEER REVIEW

The peer review history for this article is available at https://publons.com/publon/10.1002/brb3.2422

## Supporting information

Supporting information

## Data Availability

Data of this study will be made available upon request.
