# Supplementary material for: Waiting longer, feeling fatter: Effects of response delay on tactile distance estimation and confidence in females with anorexia nervosa
Source: Brain Behav. 2021 Nov 28;12(3):e2422. doi: 10.1002/brb3.2422 (PMC8933789; doi:10.1002/brb3.2422)

**Supplementary Material**

*Description Two Point Discrimination task*

The TPD task was used to assess the minimum spatial distance needed between two simultaneously presented tactile points on the skin for participants to report feeling two distinct tactile stimuli (Lundborg & Rosén, 2004; Weinstein, 1968)*.* Participants were asked to close their eyes and were presented with one or two tactile points by a calliper. These tactile points presented on the skin of the inside of the left forearm and left side of the abdomen. Participants were asked if they felt one or two points on the skin. In accordance with Weinstein (1968), the starting point was 37mm on the forearm and 33mm on the abdomen. For responses, the forced choice one up, two down staircase method was used with 5 reversals. In each trial either one (33% of the trials) or two (66%) of the trials) tactile stimuli were presented with a calliper. Only responses to the two tactile stimuli trials were used. The TPD threshold was calculated as the average of the last five correct responses. The order of the body parts was counterbalanced. It is worth noting that the reliability of this standard TPD task has been criticized due to a number of factors (Craig & Johnson, 2000), therefore for assessing basic tactile perception researchers should employ alternative methods (Tong et al., 2013).

**Table S.1.**

Demographic and clinical information AN and REC patients.

| **Participant** | **AN type** | **Other diagnoses** |
| --- | --- | --- |
| 1-AN | Restrictive |  |
| 2-AN | Restrictive |  |
| 3-AN | Restrictive |  |
| 4-AN | Restrictive | Depression, anxiety |
| 5-AN | Restrictive | Depression, anxiety |
| 6-AN | Restrictive | Depression, anxiety |
| 7-AN | Restrictive | Depression, anxiety |
| 8-AN | Restrictive | Depression, anxiety |
| 9-AN | Restrictive | Depression, anxiety |
| 10-AN | Restrictive | Depression, anxiety |
| 11-AN | Restrictive | Depression, anxiety |
| 12-AN | Binge/Purge | Depression, anxiety |
| 13-AN | Restrictive | Depression, anxiety |
| 14-AN | Restrictive | Depression, anxiety |
| 15-AN | Restrictive | Depression, anxiety |
| 16-AN | Restrictive | Depression, anxiety |
| 17-AN | Restrictive | Depression, anxiety |
| 18-AN | Restrictive | Depression, anxiety |
| 19-AN | Restrictive | Depression, anxiety |
| 20-AN | Restrictive | Depression, anxiety |
| 21-AN | OSFED | Depression, anxiety |
| 22-AN | Restrictive | Depression, anxiety |
| 23-AN | Restrictive | Depression, anxiety |
| 24-AN | Restrictive | Depression, anxiety |
| 25-AN | Restrictive | Depression, anxiety |
| 26-AN | Restrictive | Depression, anxiety |
| 27-AN | Restrictive | Depression, anxiety |
| 28-AN | Restrictive | Depression, anxiety |
| 29-AN | Restrictive | Depression, anxiety |
| 30-AN | Restrictive | Depression, anxiety |
| 1-REC | OSFED | Bulimia |
| 2-REC | Binge/Purge |  |
| 3-REC | Binge/Purge |  |
| 4-REC | Restrictive |  |
| 5-REC | Restrictive |  |
| 6-REC | Binge/Purge | Depression, anxiety |
| 7-REC | Binge/Purge | Depression, anxiety |
| 8-REC | Binge/Purge | Anxiety |
| 9-REC | Restrictive | OCD traits, anxiety, ADHD |
| 10-REC | Binge/Purge |  |
| 11-REC | Restrictive | Borderline, depression, anxiety, aspergers |
| 12-REC | Binge/Purge | Bulimia, depression |
| 13-REC | Restrictive |  |
| 14-REC | Binge/Purge | Depression, anxiety |
| 15-REC | Restrictive | OCD |
| 16-REC | Restrictive |  |
| 17-REC | Binge/Purge | Binge eating, depression, anxiety |
| 18-REC | Restrictive | Borderline, anxiety, depression, PTSD |
| 19-REC | Restrictive | Depression, anxiety |
| 20-REC | Restrictive | Anxiety |
| 21-REC | Restrictive |  |
| 22-REC | Restrictive | Anxiety |
| 23-REC | Restrictive | Depression, anxiety, chronic fatigue, fibromyalgia |
| 24-REC | Restrictive | Depression, anxiety, insomnia |
| 25-REC | Restrictive |  |
| 26-REC | Restrictive | OCD |
| 27-REC | Binge/Purge |  |
| 28-REC | Restrictive | Depression |
| 29-REC | Restrictive | Depression, anxiety |

**Table** **S.2.**

Descriptives of the TDE

| TDE | **HC**  *N* = 29 | **REC**  *N* = 29 | **AN**  *N* = 30 |
| --- | --- | --- | --- |
| Arm |  |  |  |
| Direct 50mm | -21.69 ± 20.14 | -9.13 ± 30.10 | -7.24 ± 26.67 |
| Direct 60mm | -21.74 ± 18.54 | -14.48 ± 28.81 | -19.68 ± 23.17 |
| Direct 70mm | -23.57 ± 18.46 | -16.11 ± 27.61 | -21.12 ± 21.83 |
| Delayed 50mm | -14.42 ± 20.64 | -1.10 ± 30.73 | -0.21 ± 24.90 |
| Delayed 60mm | -19.22 ± 19.00 | -8.17 ± 25.20 | -10.75 ± 19.62 |
| Delayed 70mm | -19.87 ± 20.12 | -13.10 ± 24.70 | -13.49 ± 20.44 |
| Abdomen |  |  |  |
| Direct 50mm | -13.54 ± 27.10 | -8.08 ± 32.22 | 1.40 ± 41.10 |
| Direct 60mm | -14.15 ± 23.39 | -12.75 ± 28.02 | -4.65 ± 34.12 |
| Direct 70mm | -12.35 ± 21.96 | -11.73 ± 28.66 | -6.65 ± 31.71 |
| Delayed 50mm | -13.55 ± 26.97 | 2.05 ± 32.81 | 10.41 ± 34.82 |
| Delayed 60mm | -14.86 ± 22.61 | -6.34 ± 23.98 | 2.54 ± 31.11 |
| Delayed 70mm | -14.36 ± 22.14 | -10.48 ± 22.62 | -2.11 ± 29.10 |

**Table S.3.**

Descriptives of the Confidence Ratings

| Confidence | **HC**  *N* = 26 | **REC**  *N* = 29 | **AN**  *N* = 27 |
| --- | --- | --- | --- |
| Arm |  |  |  |
| Direct 50mm | 53.87 ± 16.67 | 54.02 ± 9.34 | 52.64 ± 17.87 |
| Direct 60mm | 57.29 ± 15.01 | 56.53 ± 13.27 | 53.26 ± 17.08 |
| Direct 70mm | 59.16 ± 14.27 | 56.31 ± 12.41 | 51.88 ± 18.05 |
| Delayed 50mm | 53.73 ± 15.36 | 52.84 ± 12.53 | 50.10 ± 20.81 |
| Delayed 60mm | 57.75 ± 15.48 | 60.86 ± 22.03 | 46.50 ± 19.11 |
| Delayed 70mm | 58.82 ± 15.15 | 54.63 ± 11.04 | 50.46 ± 17.92 |
| Abdomen |  |  |  |
| Direct 50mm | 52.77 ± 17.00 | 55.72 ± 14.57 | 50.51 ± 18.69 |
| Direct 60mm | 56.81 ± 17.71 | 56.66 ± 14.02 | 54.14 ± 18.48 |
| Direct 70mm | 60.60 ± 17.97 | 57.79 ± 14.47 | 55.28 ± 16.17 |
| Delayed 50mm | 54.96 ± 19.12 | 53.97 ± 15.60 | 48.77 ± 17.42 |
| Delayed 60mm | 55.19 ± 17.16 | 54.19 ± 16.26 | 46.80 ± 17.55 |
| Delayed 70mm | 60.36 ± 16.00 | 57.36 ± 12.10 | 51.51 ± 19.17 |

**Table S.4.**

Linear mixed-model summary: TDE-D Percentage misestimation (REC dom intercepts)

| *Predictors* | *Estimates* | *S.E.* | *95% CI* | *Statistic* | *p-value* |
| --- | --- | --- | --- | --- | --- |
| (Intercept) | -10.52 | 2.22 | -14.93 – -6.12 | -4.75 | **<0.001** |
| Group [AN] | 4.70 | 3.13 | -1.53 – 10.92 | 1.50 | 0.137 |
| Group [HC] | -6.10 | 3.11 | -12.28 – 0.07 | -1.96 | 0.053 |
| BodyPart [Abdomen] | 3.36 | 0.33 | 2.70 – 4.01 | 10.09 | **<0.001** |
| Delay [0] | -2.49 | 0.33 | -3.14 – -1.84 | -7.48 | **<0.001** |
| Distance.L | -5.59 | 0.58 | -6.72 – -4.46 | -9.70 | **<0.001** |
| Distance.Q | 1.71 | 0.58 | 0.58 – 2.84 | 2.96 | **0.003** |
| Group [AN] * BodyPart [Abdomen] | 2.72 | 0.47 | 1.80 – 3.65 | 5.80 | **<0.001** |
| Group [HC] * BodyPart [Abdomen] | -0.60 | 0.47 | -1.52 – 0.32 | -1.29 | 0.198 |
| Group [AN] * Delay [0] | -1.23 | 0.47 | -2.15 – -0.31 | -2.62 | **0.009** |
| Group [HC] * Delay [0] | 1.68 | 0.47 | 0.77 – 2.60 | 3.60 | **<0.001** |
| BodyPart [Abdomen] * Delay [0] | 0.41 | 0.33 | -0.24 – 1.07 | 1.24 | 0.215 |
| Group [AN] : Distance.L | -3.08 | 0.81 | -4.67 – -1.48 | -3.79 | **<0.001** |
| Group [HC] : Distance.L | 3.74 | 0.81 | 2.15 – 5.33 | 4.62 | **<0.001** |
| Group [AN] : Distance.Q | 1.06 | 0.81 | -0.53 – 2.66 | 1.31 | 0.192 |
| Group [HC] : Distance.Q | -0.90 | 0.81 | -2.48 – 0.69 | -1.11 | 0.268 |
| BodyPart [Abdomen] : Distance.L | 1.02 | 0.58 | -0.11 – 2.15 | 1.77 | 0.077 |
| BodyPart [Abdomen] : Distance.Q | -0.21 | 0.58 | -1.34 – 0.92 | -0.37 | 0.712 |
| Delay [0] : Distance.L | 1.36 | 0.58 | 0.23 – 2.49 | 2.37 | **0.018** |
| Delay [0] : Distance.Q | -0.01 | 0.58 | -1.14 – 1.12 | -0.01 | 0.988 |
| (Group [AN] * BodyPart [Abdomen]) * Delay [0] | -0.15 | 0.47 | -1.07 – 0.77 | -0.32 | 0.746 |
| (Group [HC] * BodyPart [Abdomen]) * Delay [0] | 0.62 | 0.47 | -0.30 – 1.54 | 1.32 | 0.187 |
| Group [AN] : BodyPart [Abdomen] : Distance.L | 0.24 | 0.81 | -1.35 – 1.84 | 0.30 | 0.764 |
| Group [HC] : BodyPart [Abdomen] : Distance.L | 0.34 | 0.81 | -1.25 – 1.93 | 0.42 | 0.675 |
| Group [AN] : BodyPart [Abdomen] : Distance.Q | -1.08 | 0.81 | -2.68 – 0.51 | -1.33 | 0.184 |
| Group [HC] : BodyPart [Abdomen] : Distance.Q | 0.48 | 0.81 | -1.11 – 2.07 | 0.59 | 0.554 |
| Group [AN] : Delay [0] : Distance.L | -0.65 | 0.81 | -2.24 – 0.95 | -0.80 | 0.426 |
| Group [HC] : Delay [0] : Distance.L | -0.44 | 0.81 | -2.03 – 1.15 | -0.55 | 0.586 |
| Group [AN] : Delay [0] : Distance.Q | 0.39 | 0.81 | -1.21 – 1.98 | 0.47 | 0.636 |
| Group [HC] : Delay [0] : Distance.Q | -0.64 | 0.81 | -2.23 – 0.95 | -0.79 | 0.428 |
| BodyPart [Abdomen] : Delay [0] : Distance.L | 0.50 | 0.58 | -0.63 – 1.63 | 0.87 | 0.386 |
| BodyPart [Abdomen] : Delay [0] : Distance.Q | 0.09 | 0.58 | -1.04 – 1.22 | 0.16 | 0.876 |
| Group [AN] : BodyPart [Abdomen] : Delay [0] : Distance.L | 0.38 | 0.81 | -1.21 – 1.98 | 0.47 | 0.640 |
| Group [HC] : BodyPart [Abdomen] : Delay [0] : Distance.L | -0.53 | 0.81 | -2.12 – 1.06 | -0.65 | 0.513 |
| Group [AN] : BodyPart [Abdomen] : Delay [0] : Distance.Q | -0.32 | 0.81 | -1.91 – 1.28 | -0.39 | 0.697 |
| Group [HC] : BodyPart [Abdomen] : Delay [0] : Distance.Q | 0.44 | 0.81 | -1.15 – 2.02 | 0.54 | 0.589 |
| **Random Effects** | | | | | |
| σ^2^ | 589.22 | | | | |
| τ_00_ _Ppn_ | 431.53 | | | | |
| ICC | 0.42 | | | | |
| N _Ppn_ | 90 | | | | |
| Observations | 5333 | | | | |
| Marginal R^2^ / Conditional R^2^ | 0.055 / 0.455 | | | | |

**Table S.5.**

Linear mixed-model summary: TDE-D Confidence rating (random intercepts)

| *Predictors* | *Estimates* | *S.E.* | *95% CI* | *Statistic* | *p-value* |
| --- | --- | --- | --- | --- | --- |
| (Intercept) | 54.86 | 1.47 | 51.94 – 57.79 | 37.28 | **<0.001** |
| Group [AN] | -3.42 | 2.08 | -7.55 – 0.70 | -1.65 | 0.103 |
| Group [HC] | 2.79 | 2.08 | -1.34 – 6.91 | 1.34 | 0.183 |
| BodyPart [Abdomen] | 0.13 | 0.19 | -0.25 – 0.50 | 0.65 | 0.516 |
| Delay [0] | 0.76 | 0.19 | 0.38 – 1.14 | 3.96 | **<0.001** |
| Distance.L | 2.40 | 0.33 | 1.75 – 3.05 | 7.24 | **<0.001** |
| Distance.Q | 0.17 | 0.33 | -0.48 – 0.82 | 0.50 | 0.615 |
| Group [AN] * BodyPart [Abdomen] | -0.05 | 0.27 | -0.59 – 0.48 | -0.20 | 0.842 |
| Group [HC] * BodyPart [Abdomen] | -0.26 | 0.27 | -0.79 – 0.28 | -0.93 | 0.350 |
| Group [AN] * Delay [0] | 0.99 | 0.27 | 0.46 – 1.52 | 3.64 | **<0.001** |
| Group [HC] * Delay [0] | -0.81 | 0.27 | -1.35 – -0.28 | -2.99 | **0.003** |
| BodyPart [Abdomen] * Delay [0] | 0.09 | 0.19 | -0.29 – 0.47 | 0.47 | 0.637 |
| Group [AN] : Distance.L | -1.09 | 0.47 | -2.01 – -0.17 | -2.33 | **0.020** |
| Group [HC] : Distance.L | 1.78 | 0.47 | 0.86 – 2.70 | 3.79 | **<0.001** |
| Group [AN] : Distance.Q | 0.73 | 0.47 | -0.19 – 1.64 | 1.56 | 0.120 |
| Group [HC] : Distance.Q | -0.06 | 0.47 | -0.98 – 0.86 | -0.13 | 0.894 |
| BodyPart [Abdomen] : Distance.L | 0.72 | 0.33 | 0.07 – 1.37 | 2.17 | **0.030** |
| BodyPart [Abdomen] : Distance.Q | 0.70 | 0.33 | 0.05 – 1.35 | 2.10 | **0.035** |
| Delay [0] : Distance.L | 0.10 | 0.33 | -0.55 – 0.75 | 0.29 | 0.771 |
| Delay [0] : Distance.Q | -0.63 | 0.33 | -1.28 – 0.02 | -1.90 | 0.057 |
| (Group [AN] * BodyPart [Abdomen]) * Delay [0] | -0.03 | 0.27 | -0.56 – 0.50 | -0.10 | 0.921 |
| (Group [HC] * BodyPart [Abdomen]) * Delay [0] | -0.06 | 0.27 | -0.59 – 0.48 | -0.22 | 0.828 |
| Group [AN] : BodyPart [Abdomen] : Distance.L | 0.42 | 0.47 | -0.50 – 1.34 | 0.90 | 0.370 |
| Group [HC] : BodyPart [Abdomen] : Distance.L | 0.08 | 0.47 | -0.84 – 1.00 | 0.16 | 0.871 |
| Group [AN] : BodyPart [Abdomen] : Distance.Q | -0.76 | 0.47 | -1.67 – 0.16 | -1.61 | 0.106 |
| Group [HC] : BodyPart [Abdomen] : Distance.Q | 0.27 | 0.47 | -0.65 – 1.19 | 0.58 | 0.563 |
| Group [AN] : Delay [0] : Distance.L | -0.08 | 0.47 | -1.00 – 0.84 | -0.17 | 0.864 |
| Group [HC] : Delay [0] : Distance.L | 0.30 | 0.47 | -0.62 – 1.22 | 0.63 | 0.528 |
| Group [AN] : Delay [0] : Distance.Q | -1.01 | 0.47 | -1.92 – -0.09 | -2.15 | **0.031** |
| Group [HC] : Delay [0] : Distance.Q | 0.36 | 0.47 | -0.56 – 1.28 | 0.76 | 0.447 |
| BodyPart [Abdomen] : Delay [0] : Distance.L | 0.17 | 0.33 | -0.48 – 0.82 | 0.51 | 0.608 |
| BodyPart [Abdomen] : Delay [0] : Distance.Q | -0.39 | 0.33 | -1.04 – 0.26 | -1.18 | 0.237 |
| Group [AN] : BodyPart [Abdomen] : Delay [0] : Distance.L | 0.37 | 0.47 | -0.55 – 1.29 | 0.79 | 0.430 |
| Group [HC] : BodyPart [Abdomen] : Delay [0] : Distance.L | 0.14 | 0.47 | -0.78 – 1.06 | 0.31 | 0.760 |
| Group [AN] : BodyPart [Abdomen] : Delay [0] : Distance.Q | 0.48 | 0.47 | -0.43 – 1.40 | 1.03 | 0.302 |
| Group [HC] : BodyPart [Abdomen] : Delay [0] : Distance.Q | -0.32 | 0.47 | -1.24 – 0.60 | -0.68 | 0.495 |
| **Random Effects** | | | | | |
| σ^2^ | 188.61 | | | | |
| τ_00_ _Ppn_ | 189.34 | | | | |
| ICC | 0.50 | | | | |
| N _Ppn_ | 89 | | | | |
| Observations | 5159 | | | | |
| Marginal R^2^ / Conditional R^2^ | 0.029 / 0.516 | | | | |

**Table S.6. Pearson correlations between TDE-Q and the TDE-D.**

|  |  | Feeling about fore arm | Change evaluation forearm | Feeling about abdomen | Change evaluation abdomen |
| --- | --- | --- | --- | --- | --- |
| **AN patients** |  |  |  |  |  |
| Mean arm direct | r. | 0.152 | 0.106 | 0.167 | -0.041 |
|  | p | 0.424 | 0.578 | 0.378 | 0.829 |
| Mean arm delayed | r. | 0.271 | 0.101 | 0.247 | 0.093 |
|  | p | 0.148 | 0.596 | 0.189 | 0.625 |
| Mean abdomen direct | r. | 0.143 | 0.243 | 0.144 | 0.214 |
|  | p | 0.450 | 0.195 | 0.449 | 0.256 |
| Mean abdomen delayed | r. | 0.244 | 0.317 | 0.215 | 0.211 |
|  | p | 0.195 | 0.088 | 0.255 | 0.263 |
| **REC patients** |  |  |  |  |  |
| Mean arm direct | r. | 0.218 | -0.139 | 0.162 | 0.031 |
|  | p | 0.257 | 0.473 | 0.402 | 0.875 |
| Mean arm delayed | r. | 0.119 | -0.117 | 0.142 | 0.025 |
|  | p | 0.538 | 0.547 | 0.461 | 0.896 |
| Mean abdomen direct | r. | **406^*^** | -0.025 | 0.298 | 0.249 |
|  | p | **0.029** | 0.898 | 0.116 | 0.192 |
| Mean abdomen delayed | r. | 0.209 | 0.101 | 0.124 | 0.209 |
|  | p | 0.277 | 0.603 | 0.522 | 0.277 |
| **HC** |  |  |  |  |  |
| Mean arm direct | r. | 0.273 | -0.002 | 0.009 | 0.092 |
|  | p | 0.144 | 0.991 | 0.961 | 0.628 |
| Mean arm delayed | r. | 0.285 | 0.121 | 0.016 | -0.059 |
|  | p | 0.127 | 0.523 | 0.934 | 0.758 |
| Mean abdomen direct | r. | 0.131 | 0.029 | -0.227 | 0.049 |
|  | p | 0.482 | 0.877 | 0.220 | 0.795 |
| Mean abdomen delayed | r. | 0.120 | 0.144 | -0.251 | -0.034 |
|  | p | 0.519 | 0.439 | 0.172 | 0.854 |

Note. Critical p value is .006

**VISUALISATIONS OF RAW DATA DISTRIBUTIONS**

**Figure S.1.**

*Distribution of TDE-D percentage misestimation: Group * BodyPart*


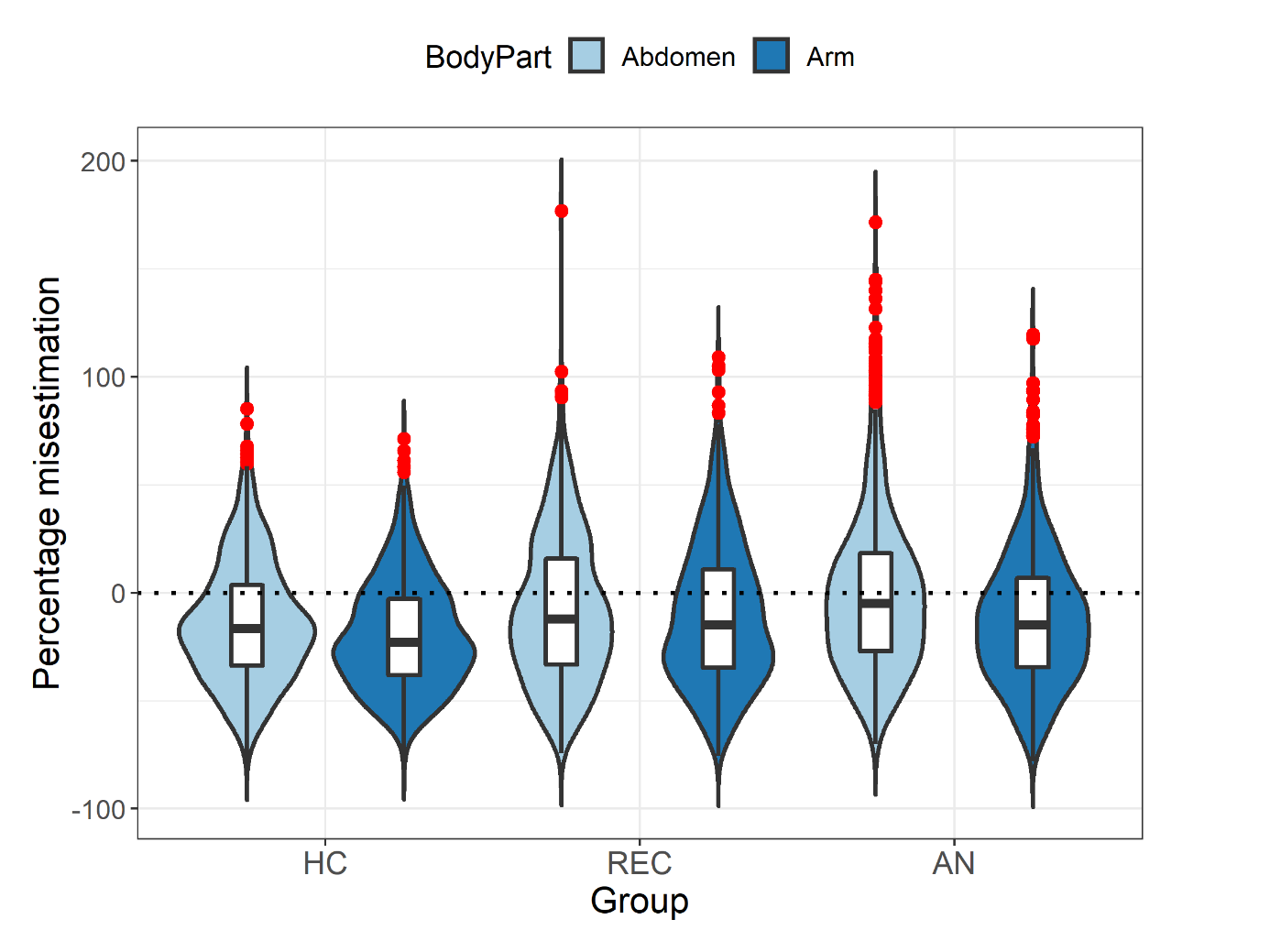


**Figure S.2.**

*Distribution of TDE-D percentage misestimation: Group * Delay*


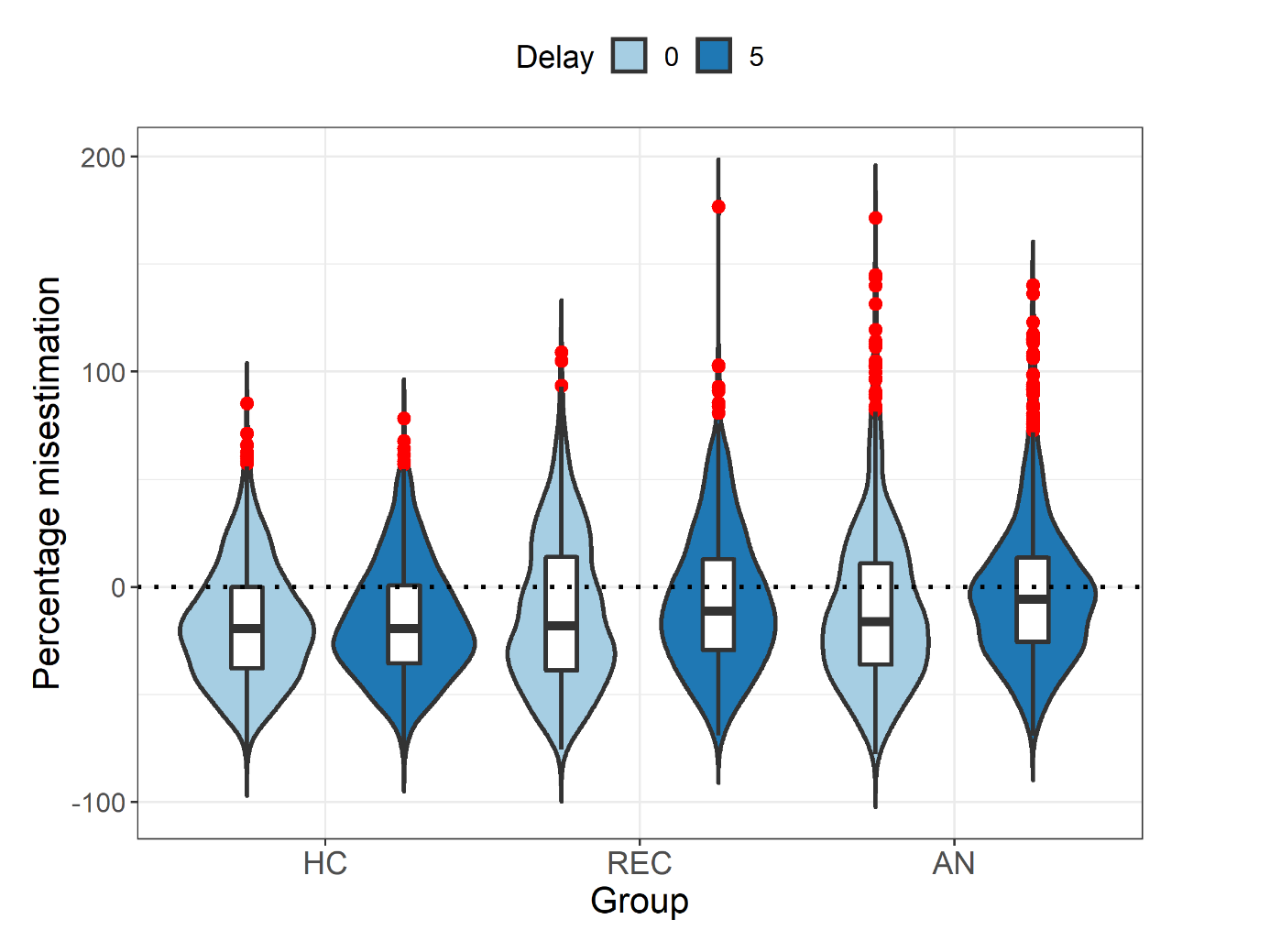


**Figure S.3.**

*Distribution of TDE-D percentage misestimation: Group * Distance*


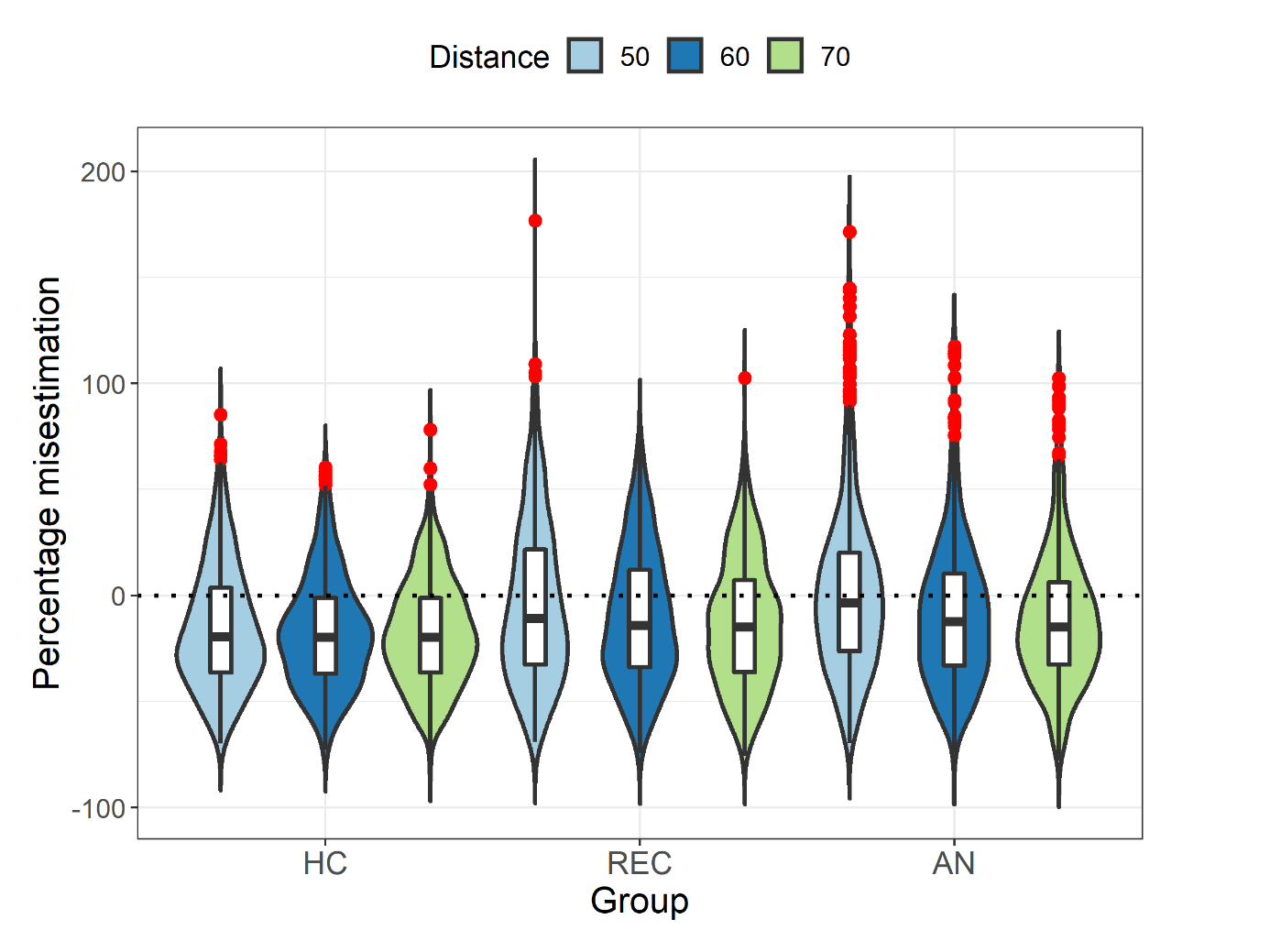


**Figure S.4.**

*Distribution of TDE-D confidence rating: Group * BodyPart*


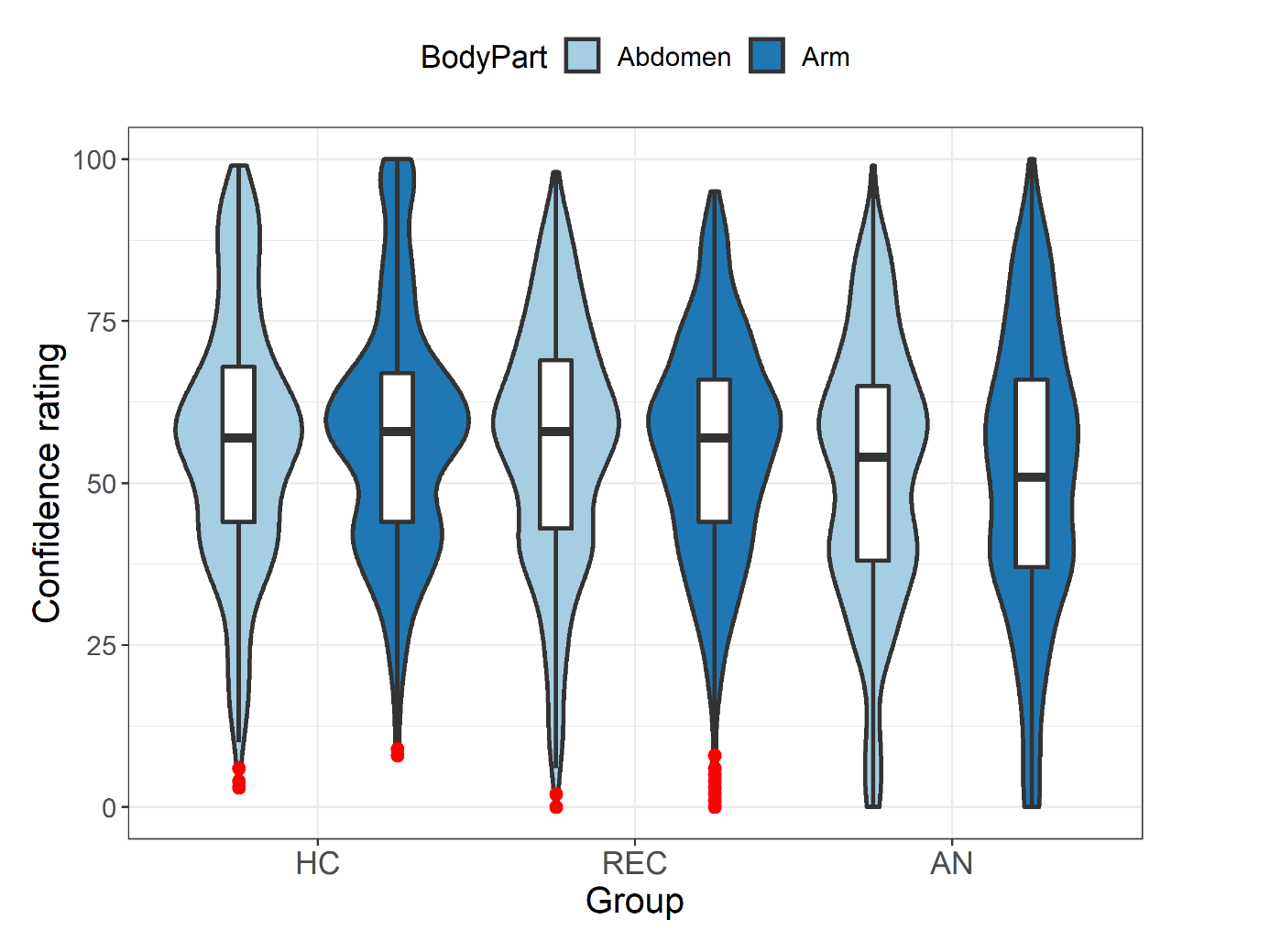


**Figure S.5.**

*Distribution of TDE-D confidence rating: Group * Delay*


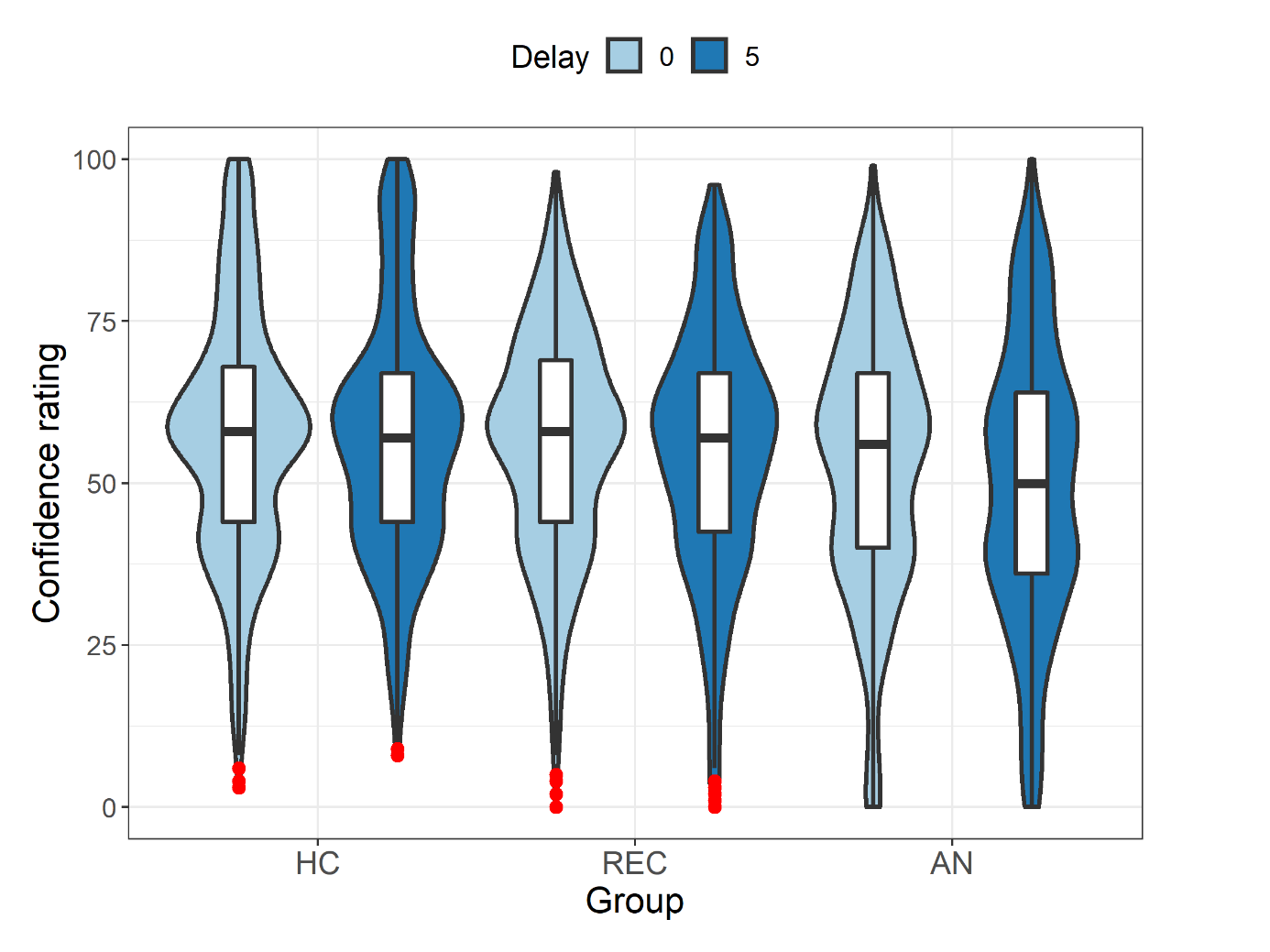


**Figure S.6.**

*Distribution of TDE-D confidence rating: Group * Distance*


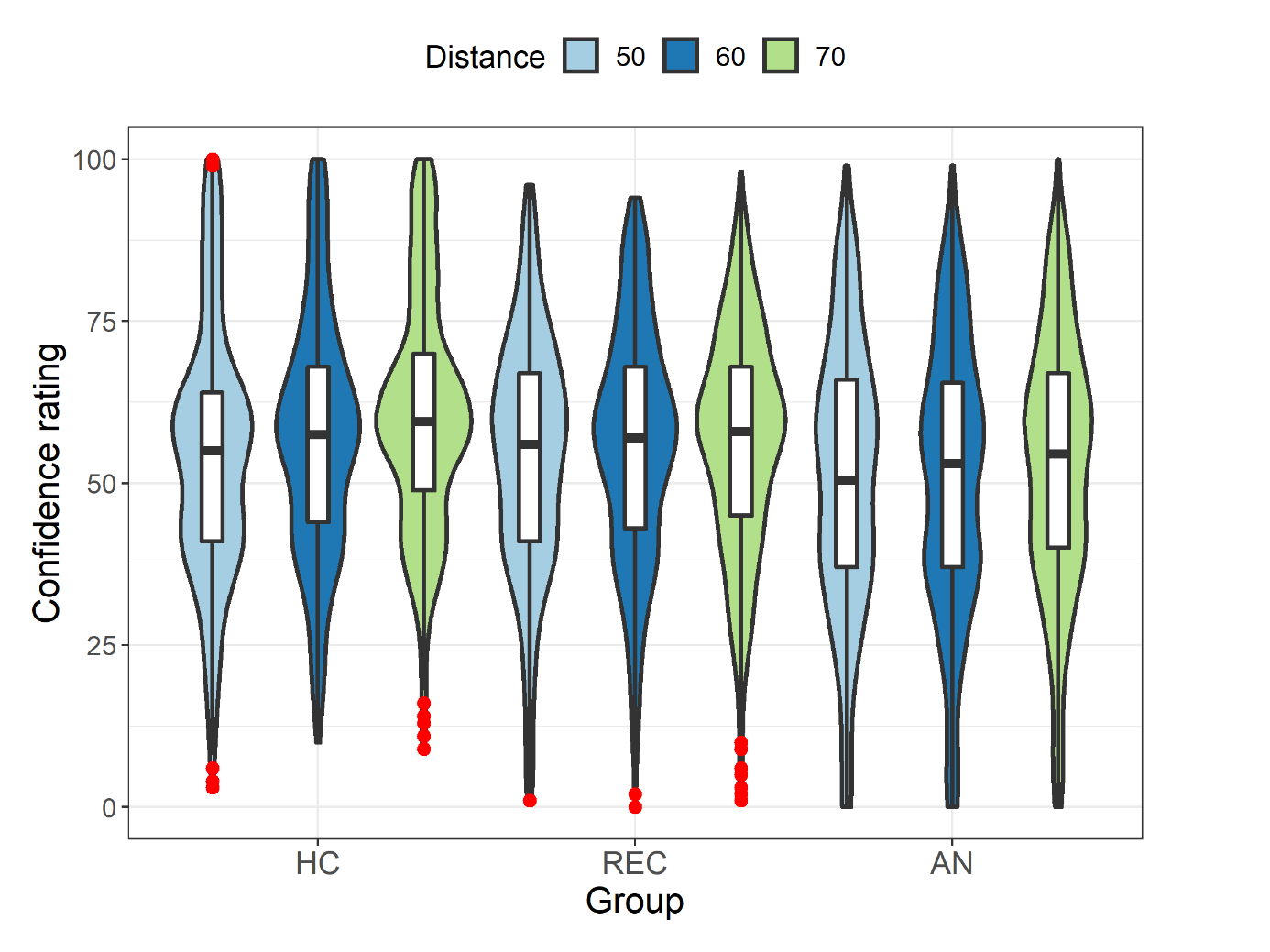


**Table S7.**

Linear mixed-model summary: TDE-D Percentage misestimation (random slopes)

|  | **Percentage misestimation** | | | | |
| --- | --- | --- | --- | --- | --- |
| *Predictors* | *Estimates* | *S.E.* | *95% CI* | *Statistic* | *p-value* |
| (Intercept) | -10.49 | 2.22 | -14.90 – -6.08 | -4.73 | **<0.001** |
| Group [AN] | 4.64 | 3.14 | -1.60 – 10.88 | 1.48 | 0.143 |
| Group [HC] | -6.06 | 3.11 | -12.25 – 0.12 | -1.95 | 0.055 |
| BodyPart [Abdomen] | 3.32 | 1.06 | 1.22 – 5.42 | 3.14 | **0.002** |
| Delay [0] | -2.49 | 0.65 | -3.78 – -1.20 | -3.83 | **<0.001** |
| Distance.L | -5.56 | 0.50 | -6.54 – -4.57 | -11.09 | **<0.001** |
| Distance.Q | 1.69 | 0.50 | 0.71 – 2.67 | 3.38 | **0.001** |
| Group [AN] * BodyPart [Abdomen] | 2.75 | 1.49 | -0.22 – 5.72 | 1.84 | 0.069 |
| Group [HC] * BodyPart [Abdomen] | -0.67 | 1.48 | -3.62 – 2.28 | -0.45 | 0.652 |
| Group [AN] * Delay [0] | -1.22 | 0.92 | -3.04 – 0.60 | -1.33 | 0.186 |
| Group [HC] * Delay [0] | 1.62 | 0.91 | -0.20 – 3.43 | 1.77 | 0.080 |
| BodyPart [Abdomen] * Delay [0] | 0.46 | 0.57 | -0.67 – 1.60 | 0.81 | 0.420 |
| Group [AN] : Distance.L | -3.07 | 0.71 | -4.45 – -1.68 | -4.33 | **<0.001** |
| Group [HC] : Distance.L | 3.74 | 0.70 | 2.36 – 5.12 | 5.31 | **<0.001** |
| Group [AN] : Distance.Q | 1.07 | 0.71 | -0.31 – 2.46 | 1.52 | 0.129 |
| Group [HC] : Distance.Q | -0.89 | 0.70 | -2.27 – 0.49 | -1.27 | 0.205 |
| BodyPart [Abdomen] : Distance.L | 1.04 | 0.50 | 0.06 – 2.02 | 2.08 | **0.038** |
| BodyPart [Abdomen] : Distance.Q | -0.21 | 0.50 | -1.19 – 0.77 | -0.42 | 0.678 |
| Delay [0] : Distance.L | 1.37 | 0.50 | 0.39 – 2.35 | 2.73 | **0.006** |
| Delay [0] : Distance.Q | -0.05 | 0.50 | -1.04 – 0.93 | -0.11 | 0.914 |
| (Group [AN] * BodyPart [Abdomen]) * Delay [0] | -0.18 | 0.81 | -1.78 – 1.43 | -0.22 | 0.828 |
| (Group [HC] * BodyPart [Abdomen]) * Delay [0] | 0.66 | 0.81 | -0.94 – 2.26 | 0.82 | 0.413 |
| Group [AN] : BodyPart [Abdomen] : Distance.L | 0.23 | 0.71 | -1.16 – 1.62 | 0.33 | 0.744 |
| Group [HC] : BodyPart [Abdomen] : Distance.L | 0.34 | 0.70 | -1.04 – 1.72 | 0.48 | 0.632 |
| Group [AN] : BodyPart [Abdomen] : Distance.Q | -1.09 | 0.71 | -2.47 – 0.30 | -1.54 | 0.124 |
| Group [HC] : BodyPart [Abdomen] : Distance.Q | 0.47 | 0.70 | -0.91 – 1.85 | 0.67 | 0.506 |
| Group [AN] : Delay [0] : Distance.L | -0.66 | 0.71 | -2.05 – 0.73 | -0.94 | 0.349 |
| Group [HC] : Delay [0] : Distance.L | -0.45 | 0.70 | -1.83 – 0.93 | -0.64 | 0.522 |
| Group [AN] : Delay [0] : Distance.Q | 0.41 | 0.71 | -0.98 – 1.80 | 0.58 | 0.563 |
| Group [HC] : Delay [0] : Distance.Q | -0.62 | 0.70 | -2.00 – 0.76 | -0.88 | 0.380 |
| BodyPart [Abdomen] : Delay [0] : Distance.L | 0.50 | 0.50 | -0.48 – 1.49 | 1.01 | 0.314 |
| BodyPart [Abdomen] : Delay [0] : Distance.Q | 0.07 | 0.50 | -0.92 – 1.05 | 0.13 | 0.896 |
| Group [AN] : BodyPart [Abdomen] : Delay [0] : Distance.L | 0.35 | 0.71 | -1.03 – 1.74 | 0.50 | 0.616 |
| Group [HC] : BodyPart [Abdomen] : Delay [0] : Distance.L | -0.55 | 0.70 | -1.93 – 0.83 | -0.78 | 0.436 |
| Group [AN] : BodyPart [Abdomen] : Delay [0] : Distance.Q | -0.31 | 0.71 | -1.70 – 1.08 | -0.44 | 0.662 |
| Group [HC] : BodyPart [Abdomen] : Delay [0] : Distance.Q | 0.45 | 0.70 | -0.94 – 1.83 | 0.63 | 0.527 |
| **Random Effects** | | | | | |
| σ^2^ | 445.75 | | | | |
| τ_00_ _Ppn_ | 435.21 | | | | |
| τ_11_ _Ppn.Delay0_ | 30.19 | | | | |
| τ_11_ _Ppn.BodyPartAbdomen_ | 92.75 | | | | |
| τ_11_ _Ppn.Delay0:BodyPartAbdomen_ | 21.70 | | | | |
| ρ_01_ | 0.09 | | | | |
|  | 0.27 | | | | |
|  | -0.01 | | | | |
| ICC | 0.56 | | | | |
| N _Ppn_ | 90 | | | | |
| Observations | 5333 | | | | |
| Marginal R^2^ / Conditional R^2^ | 0.056 / 0.581 | | | | |

**Table S.8.**

Linear mixed-model summary: TDE-D Confidence rating (Random slopes)

|  | **Confidence rating** | | | | |
| --- | --- | --- | --- | --- | --- |
| *Predictors* | *Estimates* | *S.E.* | *95% CI* | *Statistic* | *p-value* |
| (Intercept) | 54.85 | 1.47 | 51.92 – 57.78 | 37.24 | **<0.001** |
| Group [AN] | -3.44 | 2.08 | -7.57 – 0.69 | -1.66 | 0.101 |
| Group [HC] | 2.77 | 2.08 | -1.36 – 6.90 | 1.33 | 0.186 |
| BodyPart [Abdomen] | 0.12 | 0.43 | -0.74 – 0.99 | 0.28 | 0.781 |
| Delay [0] | 0.76 | 0.40 | -0.03 – 1.55 | 1.91 | 0.060 |
| Distance.L | 2.41 | 0.31 | 1.81 – 3.01 | 7.88 | **<0.001** |
| Distance.Q | 0.15 | 0.31 | -0.45 – 0.75 | 0.50 | 0.619 |
| Group [AN] * BodyPart [Abdomen] | -0.02 | 0.61 | -1.24 – 1.20 | -0.03 | 0.973 |
| Group [HC] * BodyPart [Abdomen] | -0.27 | 0.62 | -1.49 – 0.95 | -0.44 | 0.663 |
| Group [AN] * Delay [0] | 0.96 | 0.56 | -0.15 – 2.08 | 1.72 | 0.089 |
| Group [HC] * Delay [0] | -0.81 | 0.56 | -1.93 – 0.31 | -1.45 | 0.152 |
| BodyPart [Abdomen] * Delay [0] | 0.11 | 0.29 | -0.46 – 0.68 | 0.38 | 0.708 |
| Group [AN] : Distance.L | -1.06 | 0.43 | -1.91 – -0.21 | -2.45 | **0.014** |
| Group [HC] : Distance.L | 1.77 | 0.43 | 0.92 – 2.62 | 4.09 | **<0.001** |
| Group [AN] : Distance.Q | 0.72 | 0.43 | -0.13 – 1.56 | 1.67 | 0.095 |
| Group [HC] : Distance.Q | -0.05 | 0.43 | -0.90 – 0.80 | -0.12 | 0.906 |
| BodyPart [Abdomen] : Distance.L | 0.72 | 0.31 | 0.12 – 1.32 | 2.34 | **0.019** |
| BodyPart [Abdomen] : Distance.Q | 0.70 | 0.31 | 0.10 – 1.30 | 2.30 | **0.022** |
| Delay [0] : Distance.L | 0.09 | 0.31 | -0.51 – 0.69 | 0.30 | 0.768 |
| Delay [0] : Distance.Q | -0.63 | 0.31 | -1.23 – -0.03 | -2.06 | **0.040** |
| (Group [AN] * BodyPart [Abdomen]) * Delay [0] | 0.01 | 0.41 | -0.80 – 0.81 | 0.01 | 0.989 |
| (Group [HC] * BodyPart [Abdomen]) * Delay [0] | -0.05 | 0.41 | -0.86 – 0.75 | -0.13 | 0.893 |
| Group [AN] : BodyPart [Abdomen] : Distance.L | 0.43 | 0.43 | -0.42 – 1.27 | 0.98 | 0.325 |
| Group [HC] : BodyPart [Abdomen] : Distance.L | 0.06 | 0.43 | -0.79 – 0.90 | 0.13 | 0.898 |
| Group [AN] : BodyPart [Abdomen] : Distance.Q | -0.76 | 0.43 | -1.60 – 0.09 | -1.75 | 0.079 |
| Group [HC] : BodyPart [Abdomen] : Distance.Q | 0.27 | 0.43 | -0.58 – 1.12 | 0.63 | 0.531 |
| Group [AN] : Delay [0] : Distance.L | -0.08 | 0.43 | -0.93 – 0.77 | -0.19 | 0.851 |
| Group [HC] : Delay [0] : Distance.L | 0.30 | 0.43 | -0.55 – 1.15 | 0.70 | 0.483 |
| Group [AN] : Delay [0] : Distance.Q | -1.00 | 0.43 | -1.85 – -0.16 | -2.32 | **0.020** |
| Group [HC] : Delay [0] : Distance.Q | 0.36 | 0.43 | -0.49 – 1.21 | 0.82 | 0.410 |
| BodyPart [Abdomen] : Delay [0] : Distance.L | 0.17 | 0.31 | -0.43 – 0.77 | 0.55 | 0.582 |
| BodyPart [Abdomen] : Delay [0] : Distance.Q | -0.39 | 0.31 | -0.98 – 0.21 | -1.26 | 0.206 |
| Group [AN] : BodyPart [Abdomen] : Delay [0] : Distance.L | 0.37 | 0.43 | -0.47 – 1.22 | 0.87 | 0.386 |
| Group [HC] : BodyPart [Abdomen] : Delay [0] : Distance.L | 0.12 | 0.43 | -0.72 – 0.97 | 0.29 | 0.774 |
| Group [AN] : BodyPart [Abdomen] : Delay [0] : Distance.Q | 0.47 | 0.43 | -0.37 – 1.32 | 1.10 | 0.273 |
| Group [HC] : BodyPart [Abdomen] : Delay [0] : Distance.Q | -0.30 | 0.43 | -1.15 – 0.55 | -0.70 | 0.484 |
| **Table S9.**  Random Effects | | | | | |
| σ^2^ | 160.30 | | | | |
| τ_00_ _Ppn_ | 189.76 | | | | |
| τ_11_ _Ppn.Delay0_ | 10.91 | | | | |
| τ_11_ _Ppn.BodyPartAbdomen_ | 13.58 | | | | |
| τ_11_ _Ppn.Delay0:BodyPartAbdomen_ | 4.25 | | | | |
| ρ_01_ | -0.08 | | | | |
|  | 0.15 | | | | |
|  | -0.01 | | | | |
| ICC | 0.57 | | | | |
| N _Ppn_ | 89 | | | | |
| Observations | 5159 | | | | |
| Marginal R^2^ / Conditional R^2^ | 0.030 / 0.585 | | | | |

**Figure S.7.**

*TDE Group*Bodypart*Delay*


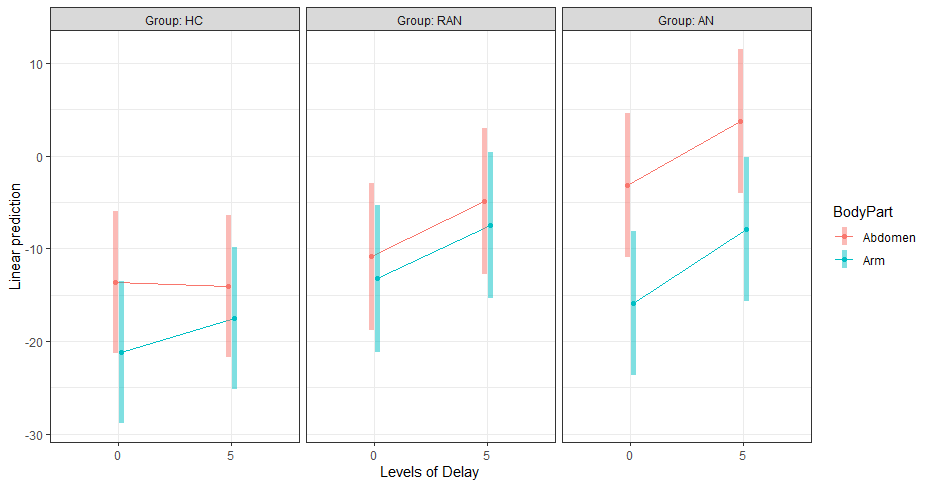

Supplement: Supplementary file 1 — Supporting information [file BRB3-12-e2422-s001.docx]
